# Resensitization of Akt Induced Docetaxel Resistance in Breast Cancer by ‘Iturin A’ a Lipopeptide Molecule from Marine Bacteria *Bacillus megaterium*

**DOI:** 10.1038/s41598-017-17652-z

**Published:** 2017-12-11

**Authors:** Goutam Dey, Rashmi Bharti, Anjan Kumar Das, Ramkrishna Sen, Mahitosh Mandal

**Affiliations:** 1 School of Medical Science & Technology, Indian Institute of Technology Kharagpur, Kharagpur, 721302 India; 2 Department of Pathology, Calcutta National Medical Collage, Kolkata, 70014 West Bengal India; 3 Department of Biotechnology, Indian Institute of Technology Kharagpur, Kharagpur, 721302 India

## Abstract

Development of the resistance is the major problem in cancer therapy. Docetaxel is a taxol alkaloid that is frequently used in metastatic breast cancer. However, resistance often limits the usefulness of this drug in many breast cancer patients. Manipulation of resistant cells to re-sensitize to the therapeutic effect of docetaxel is current strategy to overcome this problem. Here, we have introduced ‘Iturin A’ as a potent chemosensitizer in docetaxel resistant breast cancer cells. Combination of Iturin A and docetaxel treatment significantly hampered the proliferation of docetaxel resistant MDA-MB-231 and MDA-MB-468 breast cancer cells. Cell cycle analysis also showed massive amount of apoptotic population (Sub G0/G1) in combination therapy. A number of apoptotic and anti-apoptotic proteins were significantly altered in dual drug treated groups. Caspase 3 dependent cell death was observed in dual treatment. Molecular mechanism study showed that over-expression of Akt and its downstream signaling pathway was associated with docetaxel resistance. Iturin A significantly reduced Akt signaling pathway in resistant cells. This mechanistic action might be the reason behind the chemo-sensitization effect of Iturin A in docetaxel resistant breast cancer cells. In conclusion, Iturin A resensitized the resistant breast cancer cells to docetaxel therapy by inhibiting Akt activity.

## Introduction

Breast cancer is the most frequently disease diagnosed worldwide. Therapeutic strategy depends on expression of a number of receptors including estrogen receptor, progesterone receptor and human growth factor receptor 2. The clinical benefits of hormonal therapy, chemotherapy and immunotherapy are highly co-related with the expression of above receptors.

Docetaxel is a FDA approved semi-synthetic taxol alkaloid molecule. It is used as 1^st^ line chemotherapeutic agent in metastatic breast cancer therapy. It displays cell cycle arrest and apoptosis in cancer cells by stabilizing and preventing depolymerization of microtubules. However, development of docetaxel resistance is major clinical problem in different cancers including breast cancer patients^1^. Activation of a number of survival signaling pathways has been reported to promote resistant phenotype in cancer cells in response to docetaxel treatment. Other factors are activation of P-glycoprotein, inhibition of apoptotic mechanism, metabolic alteration, changes in microtubule structure and binding efficiency of docetaxel to the microtubules^2^. P-glycoprotein or multidrug resistant protein 1 (MDR 1) is the major contributing factor for chemotherapeutic insensitivity.

Akt signaling plays a critical role in cancer associated phenomena including tumorigenesis, proliferation, invasion, metastasis and angiogenesis. Activated Akt pathway is also responsible for development of resistance in cancer cells to various chemotherapeutics drugs^3–6^. Moreover, Akt has been found to have positive co-relation with P-glycoprotein that is involved in drug efflux and multidrug resistance in cancer cells^7^. In previous report, Akt was found to be up-regulated in docetaxel resistant cancer cells^8^. So, Akt inhibition can lead to development of new therapeutics strategy to overcome chemoresistance. Some earlier report showed that some specific Akt inhibitors can resensitize cancer cells to various chemotherapeutics agents^9,10^. In breast cancer model Akt is the critical mediator for insensitivity of chemotherapeutic agents. High level of Akt in breast cancer cells was co-related with enhanced resistance to chemotherapeutic agents and tumor with more Akt expression are susceptible to recurrence and relapse^11^.

Microbial lipopeptides represent a unique class of pharmacologically active molecules^12^. In pharmaceutical application, lipopeptides particularly daptomycin, caspofungin and viscosin are reported to have potential anti-bacterial, anti-fungal and anti-viral activities^13^. Recently, it was found that lipopeptides are also effective in multidrug resistant bacteria including methicillin-resistant *Staphylococcus aureus* and penicillin resistant *Streptococcus pneumonia*, gram negative bacteria and extremely drug resistant clinically *isolates* ^13–15^. In our previous findings we isolated ‘Iturin A’ from marine bacteria *Bacillus megaterium* and tested its anti-tumor efficacy in various *in vitro* and *in vivo* models. It displayed anti-cancer efficacy by inhibiting Akt signaling pathway^16,17^. However, the effect of Iturin A on resistance cancer cells was not previously documented. In the current investigation, we systematically tested the potential efficacy of Iturin A in docetaxel resistant cancer cells. Our hypothesis was that due to Akt inhibitory effect, Iturin A could reverse the insensitivity of docetaxel resistant breast cancer cells.

## Results

### Generation and establishment of docetaxel resistant cells

**Figure 1:**
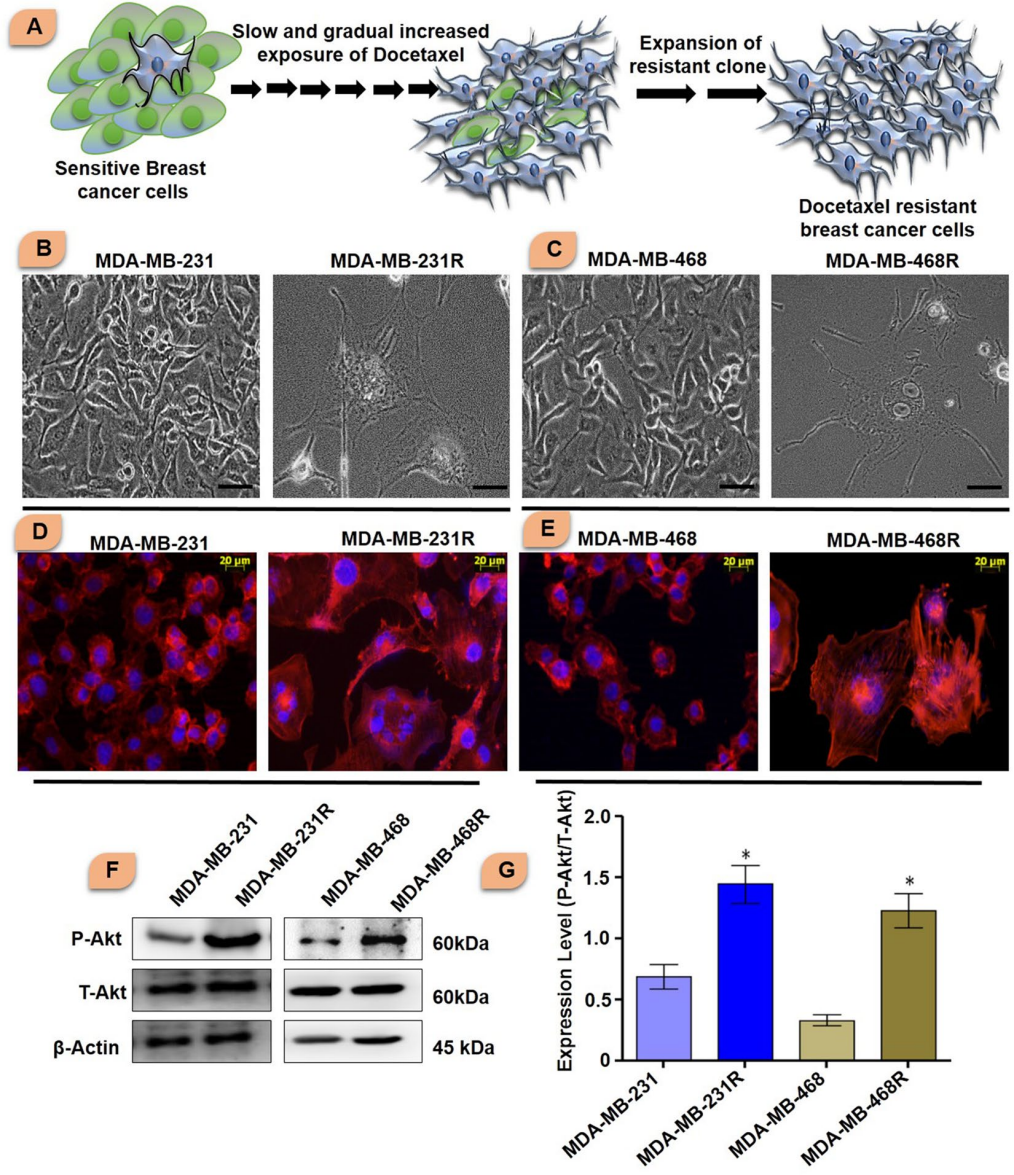
(**A**) Graphical representation of generation of resistant breast cancer cells from sensitive breast cancer cells. Docetaxel sensitive MDA-MB-231 and MDA-MB-468 cells were cultured and treated with low dose of docetaxel and then dose was increased gradually to generate resistant breast cancer cells. (**B** and **C**) Phase contrast images of sensitive and resistant breast cancer cells. Scale bar represents 100 µm. (**D** and **E**) Actin organization of sensitive and resistant breast cancer cells was shown in figure. Sensitive cells were small in size, normal actin organization. The actin organization of resistant cells was elongated and parallel orientation of actin filaments. (**F** and **G**) Akt expression in sensitive and resistant breast cancer cells (Blots were cropped and presented). The significance level was represented by *P < 0.05, **P < 0.01, ***P < 0.001.

Sensitive MDA-MB-231 and MDA-MB-468 breast cancer cells were treated with gradual increasing dose of docetaxel to establish docetaxel resistant cell lines. Initially cells were treated with 20 nM of docetaxel and further cells were treated with increasing dose of docetaxel and subsequently passaged for one year (Fig. 1A).

### Morphological features of sensitive and resistant cells

Prominent morphological changes were observed due to development of docetaxel resistance in breast cancer cells. The comparative morphology of MDA-MB-231, MDA-MB-468, MDA-MB-231R and MDA-MB-468R cells was shown in Fig. 1B and C. The morphology of sensitive cells was small size, well organized nucleus, and absence of vesicles. On the other hand resistant breast cancer cells displayed large cellular size, irregular shape, multiple nucleuses and increasing number of large vesicles.

### Cytoskeleton rearrangement in resensitized breast cancer cells

Cytoskeleton rearrangement was determined by rhodamine phalloidin staining. Sensitive cells displayed a well-organized actin filament without stress fiber. Docetaxel resistant cells showed parallel actin filaments with stress fiber. Treatment with Iturin A and docetaxel caused prominent actin reorganization, cellular shrinkage and collapse of actin filaments in resistant breast cancer cell lines (Fig. 1D and E).

### Comparative protein expression in sensitive and resistant breast cancer cells

We performed western blot analysis to check the level of different proteins associated with resistant development process. At first Akt level was checked (Fig. 1F and G). The expression of P-Akt was observed to be significantly high in resistant MDA-MB-231R and MDA-MB-468R cells compared to sensitive cells. Total Akt level was found almost equal in both sensitive and resistant cells.

### Determination of IC_50_ doses in breast cancer cells

**Figure 2:**
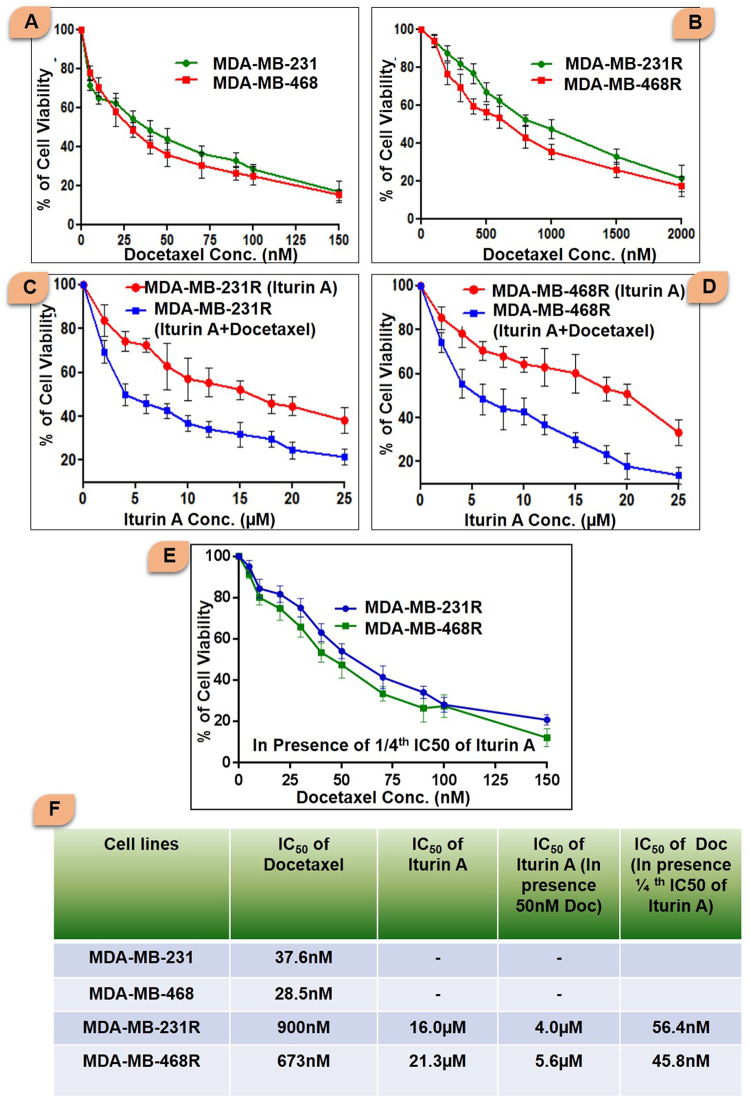
MTT assay of sensitive and resistant breast cancer cells treated with various treatment strategies. (**A**) Sensitive MDA-MB-231 and MDA-MB-468 cells were treated with various doses of docetaxel for 48 h and MTT assay was performed. (**B**) MTT assay was performed to determine the IC_50_ values of docetaxel in resistant MDA-MB-231R and MDA-MB-468R cells. (**C**) MDA-MB-231R cells were treated with Iturin A or combination of Iturin A along with 50 nM docetaxel for 48 h. The IC_50_ values Iturin A were determined. (**D**) MDA-MB-231R cells were treated with Iturin A or combination of Iturin A along with 50 nM docetaxel. The IC_50_ values Iturin A were determined. (**E**) MTT assay to determine IC_50_ of Docetaxel in presence of Iturin A (1/4th IC_50_ dose of Iturin A) in resistant cells. (**F**) IC_50_ values of Docetaxel and Iturin A were shown in various treatment strategies.

The sensitivity of docetaxel in sensitive and resistant breast cancer cells was determined by conventional MTT assay (Fig. 2). The IC_50_ values of docetaxel in sensitive and resistant cells were presented in Fig. 2A and B. The IC_50_ value of Iturin A and docetaxel in resistant cells was also reported in Fig. 2F. MTT assay was performed to determine IC50 dose of docetaxel in presence of Iturin A (Fig. 2E) in resistant cells. Further, MTT assay was performed in resistant cells treated with different dose of Iturin A in absence or presence of 50 nM dose of docetaxel (Fig. 2C and D). In this combination, the IC50 values of Iturin A were 4.0 µM and 5.6 µM in MDA-MB-231R and MDA-MB-468R cells (Fig. 2F). Based on the result from MTT assay, we selected the dose of Iturin A and docetaxel for further experiments.

### Iturin A potentiated docetaxel mediated toxicity

**Figure 3:**
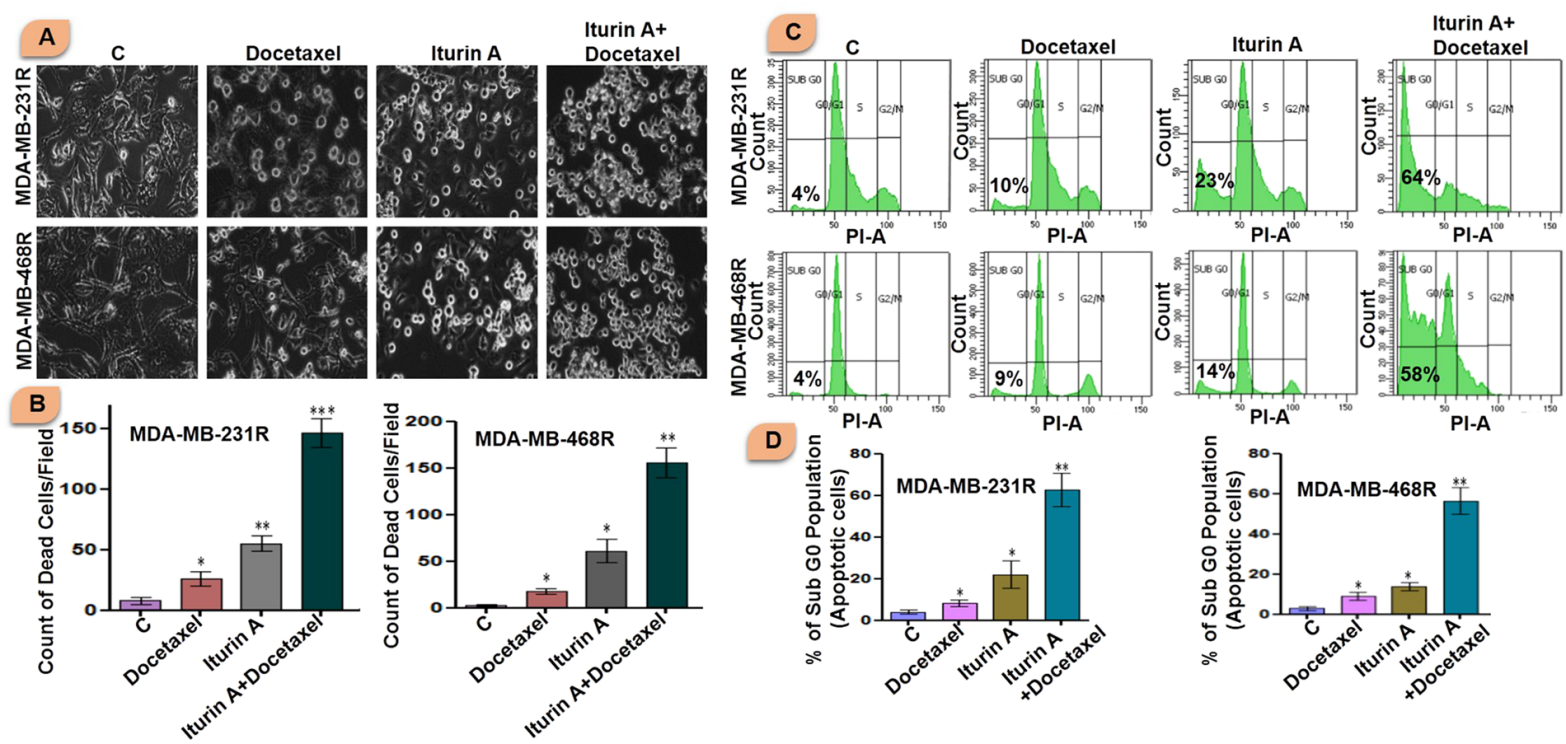
(**A** and **B**) Phase contrast images resistant breast cancer cells treated with docetaxel and/or Iturin A for 48 h. Apoptotic cells were counted in each group. Resistant MDA-MB-231R and MDA-MB-468R cells were treated with docetaxel and/or Iturin A for 48 h. Bar graph represented arbitrary number apoptotic dead cells in MDA-MB-231R and MDA-MB-468R. (**C** and **D**) Flow cytometric analysis of resistant breast cancer cells treated with docetaxel and/or Iturin A for 48 h to determine DNA content based quantitative apoptotic effect. Bar graph represented apoptotic population (Sub G_0_) cells in MDA-MB-231R and MDA-MB-468R. In all experiments, cells were treated with Iturin A (4 µM for MDA-MB-231R and 5.6 µM for MDA-MB-468R) and docetaxel (50 nM in both resistant cells) in specific doses. The significance level was represented by *P < 0.05, **P < 0.01, ***P < 0.001.

Resistant cells were treated with Iturin A and/or docetaxel for 48 h and microscopic observation was recorded by phase contrast microscope (Fig. 3A and B). Docetaxel caused very few cellular deaths in both resistant cells. Iturin A also caused slight cellular death. Interestingly, huge number of dead cells (Characterized by loss of lamellipodia/filopodia, cell rounding and shrinkage of cells and detachment of cells) was observed in combination treatment of docetaxel and Iturin A groups.

### Quantification of apoptotic cell death by flow cytometry

Resistant MDA-MB-231R and MDA-MB-468R cells were treated with Iturin A and/or docetaxel. The combination of these two agent caused huge apoptotic cellular death in resistant cells. Single treatment with Iturin A or docetaxel displayed slight apoptotic effect in both cell lines (Fig. 3C and D).

### Detection of early apoptosis in resistant breast cancer cells

**Figure 4:**
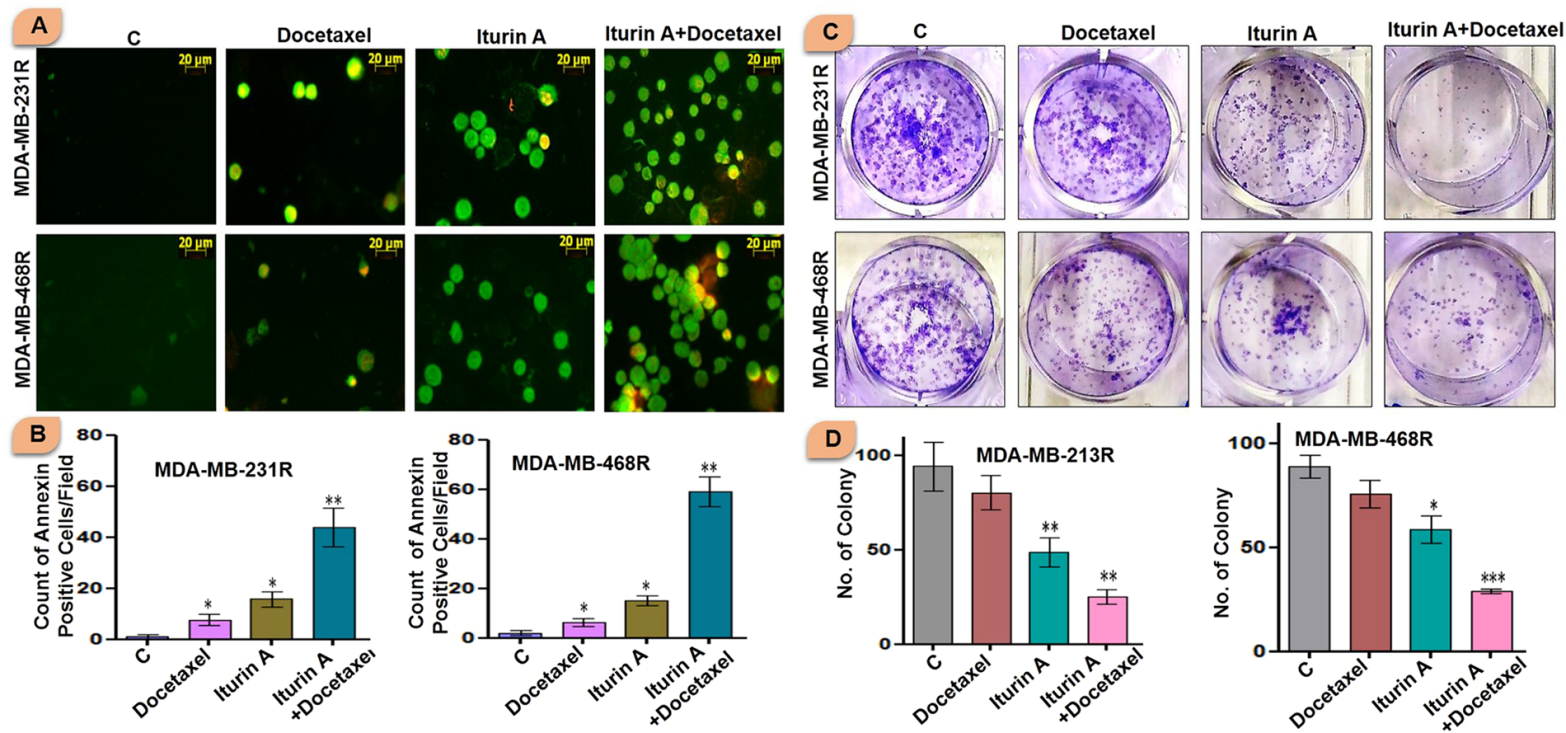
(**A** and **B**) Annexin-FITC/PI staining of resistant breast cancer cells treated with docetaxel and/or Iturin A for 48 h for detection of early apoptotic effect. Bar graph represented arbitrary number early apoptotic MDA-MB-231R and MDA-MB-468R cells. (**C** and **D**) Colony formation assay of resistant breast cancer cells MDA-MB-231R and MDA-MB-468R treated with docetaxel and/or Iturin A for 48 h to analyze cell reproductive death. Bar graph represented number of colony in different groups of MDA-MB-231R and MDA-MB-468R. In all experiments, cells were treated with Iturin A (4 µM for MDA-MB-231R and 5.6 µM for MDA-MB-468R) and docetaxel (50 nM in both resistant cells) in specific doses. The significance level was represented by *P < 0.05, **P < 0.01, ***P < 0.001.

Externalization of phosphatidylserine was detected in treated resistant breast cancer cells. This biochemical phenomenon is associated with early apoptotic event. Our study showed that few annexin positive cells (green florescent) were present in Iturin A treated group. In docetaxel treated group less number of annexin positive cells was present. However in combined treated group, significantly high numbers of annexin positive cells were observed indicating elevated apoptotic induction due to resensitization effect of Iturin A (Fig. 4A and B).

### Iturin A and docetaxel significantly suppressed the colony formation

Cell reproductive death was detected by conventional colony formation assay. A large number of colony was observed in control cells. Iturin A treatment showed slight inhibition of number of colony formation. Further, docetaxel treatment had no significant effect in colony formation. Significantly less number of colonies was observed in combined treated MDA-MB-231R and MDA-MB-468R (Fig. 4C and D).

### Western blot experiment displayed modulation of apoptosis regulatory proteins

**Figure 5:**
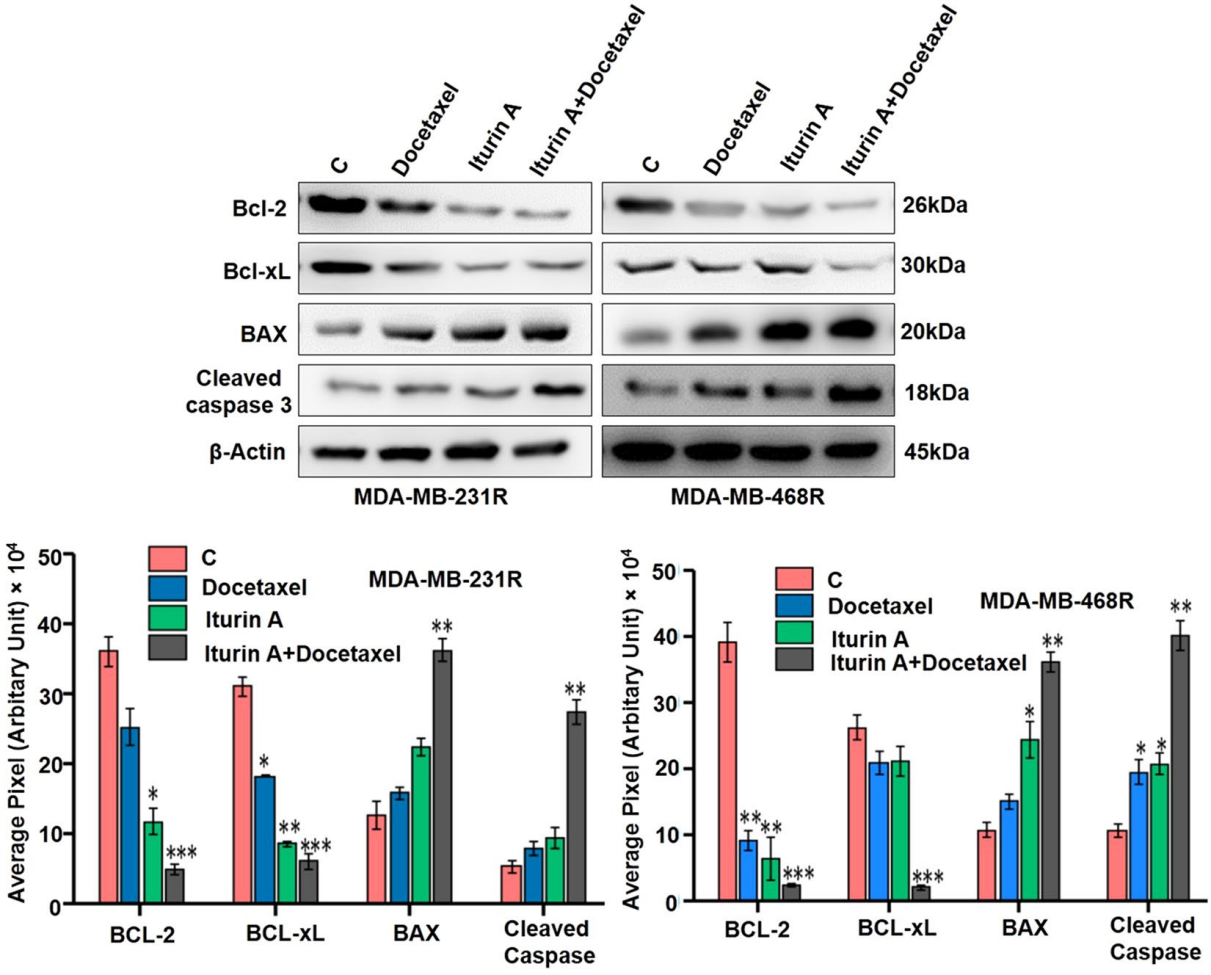
Western blot analysis of apoptosis related proteins. (**A**) Western blot analysis of MDA-MB-231R and MDA-MB-468R treated with docetaxel and/or Iturin A for 48 h (Blots were cropped and presented). (**B**) Bar graph represented densitometric plot of protein expression in various groups in MDA-MB-231R and MDA-MB-468R. The significance level was represented by *P < 0.05, **P < 0.01, ***P < 0.001.

Resistant breast cancer cells were treated with Iturin A, docetaxel or combination. Expression of various proteins was observed. Expression of anti-apoptotic proteins (Bcl-2 and Bcl-xL) were significantly down regulated in combination treated group. Apoptotic protein, Bax was significantly up-regulated in combined treated group in both MDA-MB-231R and MDA-MB-468R cells. Bcl-2/Bax ratio was significantly reduced in dual drug treated groups. Caspase 3 is the critical mediator of apoptosis phenomenon. In our experiment cleavage of caspase 3 was prominent in resensitized cells indicating huge cell death in the combination group (Fig. 5A and B).

### Inhibition of Akt signaling resensitized the resistant cells to docetaxel

**Figure 6:**
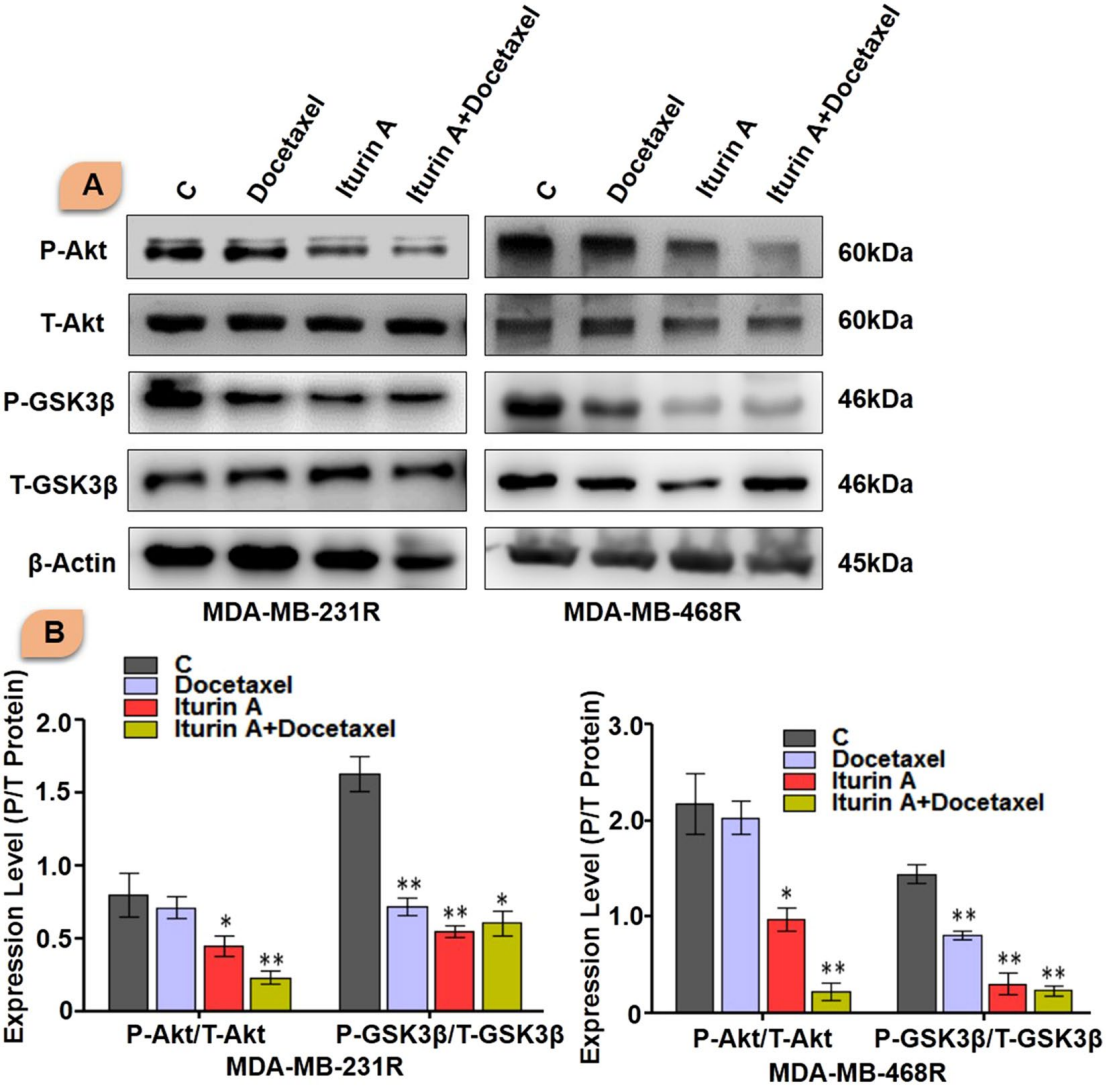
Western blot analysis of resistant cells. (**A**) Western blot analysis of MDA-MB-231R and MDA-MB-468R treated with docetaxel and/or Iturin A for 48 h (Blots were cropped and presented). (**B**) Bar graph represented densitometric plot of protein expression in various groups in MDA-MB-231R and MDA-MB-468R. The significance level was represented by *P < 0.05, **P < 0.01, ***P < 0.001.

Next we evaluated expression of P-Akt and its downstream signaling. Iturin A slightly inhibited P-Akt expression and docetaxel treatment fails to inhibit P-Akt in resistant cells. Combination of Iturin A and docetaxel treatment significantly suppressed P-Akt expression. Expression level of total Akt protein was not significantly change among various groups. GSK3β is downstream protein of Akt signaling. We also tested phosphorylation status of GSK3β. Like P-Akt, similar kind of results was observed in P-GSK3β (Fig. 6A and B).

## Discussions

The menace of chemoresistance is the potential drawback during the clinical management of multiple cancers including breast cancer. During the early stage of treatment, chemotherapeutic drugs may be effective but gradually they display poor clinical response due to acquired resistance of cancer cells^18^. Some chemotherapeutic drugs are also less effective to cancer owing to inherent resistance of cancer cells^19^. Numerous molecular drivers in cancer cells were discovered previously expanding the knowledge domain of chemoresistance^20^. These findings enable the scientist to discover novel therapeutic approaches to overcome the chemoresistance. Docetaxel is currently drug of choice for treatment of locally advanced and metastatic breast cancer^21^. In addition, docetaxel is the drug of choices for triple negative breast cancer. So, in our study we selected highly invasive and triple negative breast cancer cells MDA-MB-231 and MDA-MB-468. Many cancer patients are unresponsive or less responsive to docetaxel therapy. Resensitization of resistant cancer cells with other agent to chemotherapeutic drug is established strategy. In this current investigation, we evaluated the effect of Iturin A in docetaxel resistant cancer cells.

Initially, we generated the resistant cells from sensitive cells (Fig. 1A). Characteristics cellular arrangement was observed in resistant cells by phase contrast (Fig. 1B and C) and rhodamine phalloidin staining (Fig. 1D and E). Large and multiple number of vesicles inside the resistant cells were observed. In addition to this, actin reorganization, increased cellular size and presence of multinucleations were also observed in docetaxel resistant cells. These altered morphological features were previously reported in resistant cancer cells including multiple vesicles, large and multi-neucleated cells^22,23^. Presence of large number of vesicles may prevent the cells from the lethal effects of cytotoxic drugs by employing different mechanisms^24,25^. Akt has prominent role in tumorigenesis, cell survival, radioresistance and chemoresistance^3,26^ in multiple cancers. Expression of P-Akt was evaluated in sensitive and resistant cells. Our result showed that P-Akt expression was elevated in resistant MDA-MB-231R and MDA-MB-468R cells compare to sensitive cells (Fig. 1F and G). Inhibition of Akt is accepted strategy to overcome resistance in cancer cell^27^. The sensitivity of Iturin A was tested in resistant cells by MTT assay (Fig. 2). The IC_50_ value of docetaxel was determined in sensitive as well as resistant cells (Fig. 2). In resensitization assay, 50 nM docetaxel showed 50% cellular proliferation in presence of 4.0 and 5.6 µM of Iturin A in MDA-MB-231R and MDA-MB-468R cells. These finding clearly indicated the reversal effect of Iturin A in MDA-MB-231R and MDA-MB-468R cells (Fig. 2). Phase contrast microscopy was performed to check effect of Iturin A on MDA-MB-231R and MDA-MB-468R cells resensitization. Single treatment with Iturin A or docetaxel displayed very less apoptotic effect on MDA-MB-231R and MDA-MB-468R cells. However, the combination of these two agents showed significant level of apoptosis in resistant cells (Fig. 3A and B). Next, flow cytometry based cell cycle analysis was performed. Similar with previous result, individual treatment showed very less apoptotic population and combined treatment caused drastic apoptotic population in resistant cells (Fig. 3C and D). Externalization of phosphatidylserine is the critical marker for detection of early apoptotic events^28^. Here annexin/PI staining was performed to detect early apoptosis. Enormous numbers of early apoptotic cells (green florescent) were observed in combination treatment compare to individual treated group (Fig. 4A and B). Next we have performed colony forming capacity of resistant cells treated with Iturin A or docetaxel. Generally, colony formation assay determines cell reproductive death of cancer cells by anti-cancer agents measuring the effectiveness of chemotherapy^29^. Combined treatment displayed very low number of colonies reflecting potent cytotoxic action of this combination on reproductive ability of cancer cells (Fig. 4C and D). Apoptosis analysis at protein level was checked by western blot analysis. Bcl-2 protein is the critical component of tumor associated apoptosis event. Overexpression of Bcl-2 protein can promote proliferation tumorigenesis and inhibition of apoptosis in multiple cancers. In our findings, Iturin A and docetaxel potently suppress Bcl-2 expression in combination (Fig. 5A and B). Another apoptosis related important protein is Bax. Bax down regulation play a critical role in cancer progression^30^. Apoptosis inducing chemotherapeutic drugs often up-regulate Bax expression in cancer cells^31^. In our study, significant Bax up-regulation was noticed in resensitized cells treated with Iturin A in combination with docetaxel (Fig. 5). Caspase activation is the necessary part of biological process of apoptosis^32^. Here caspase 3 activations were found prominent in combination therapy in resistant MDA-MB-231R and MDA-MB-468R cells (Fig. 5). Akt signaling pathway has been reported to promote cell survival, inhibition of apoptosis, resistance to multiple chemotherapeutic drugs like cisplatin, carboplatin, doxorubicin, 5-flurouracil, cyclophosphamide, paclitaxel and docetaxel^4,33–38^. Inhibition of Akt signaling can resensitize the cancer cells. Our study showed that inhibition of Akt by Iturin A treatment resensitized the resistant cells to docetaxel treatment. Expression of phospho Akt was reduced in Iturin A treated group. Docetaxel treatment was unable to inhibit P-Akt in MDA-MB-231R and MDA-MB-468R cells. However, drastic change of P-Akt expression was noticed in Iturin A along with docetaxel treated groups (Fig. 6). The downstream protein GSK3β is major Akt substrate. Phosphorylation state of GSK3β was also drastically downregulated in resensitized cells treated with Iturin A and docetaxel combination (Fig. 6).

**Figure 7:**
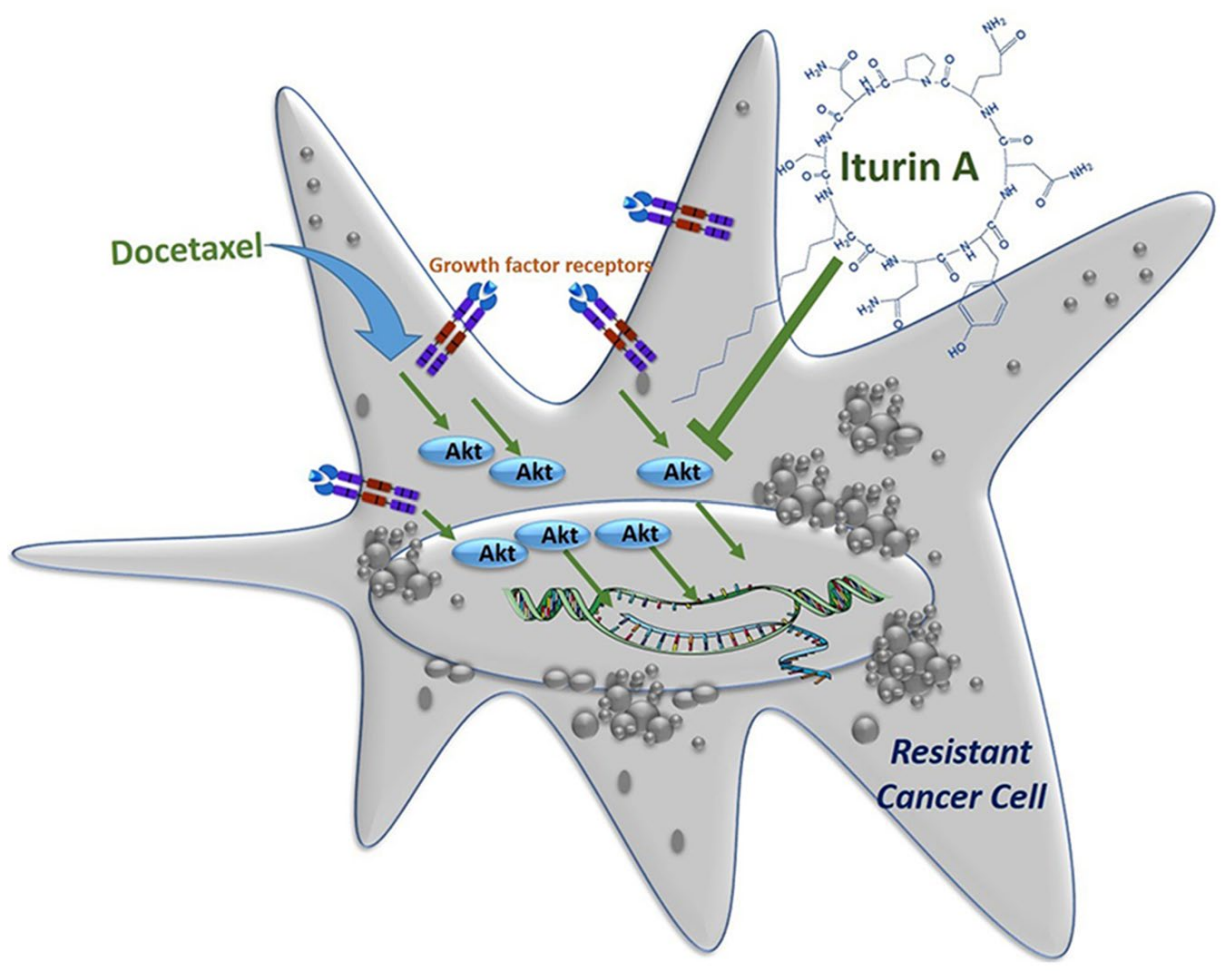
Proposed mechanism of action of Iturin A in inhibiting resistant cancer cells through suppressing Akt activity.

Our findings offered a novel approach to overcome chemoresistance that is a major clinical problem in chemotherapy. Docetaxel treatment is the critical part of standard chemotherapeutic regimen of breast cancer therapy. However, insensitivity or resistance to docetaxel therapy is the challenging drawback for successful cancer management. Akt is frequently over-expressed in resistant cell population. In our study, Iturin A inhibited Akt and restored the sensitivity of resistant MDA-MB-231R and MDA-MB-468R breast cancer cells to docetaxel therapy (Fig. 7).

## Materials and Methods

### Chemical reagents

Docetaxel was purchased from Sigma-Aldrich. Stock solution of docetaxel was prepared in DMSO and stored in −20 °C. Further, working solution was prepared in incomplete DMEM medium. Iturin A was dissolved in mili Q water and pH was adjusted to 7.0. A number of antibodies were procured as following: Bcl-2, Bcl-XL, MCL-1, Bax and caspase-3, (Cell Signaling, Beverly, MA, USA). β-actin, mouse IgG and rabbit IgG (Sigma-Aldrich, St. Louis, MO, USA). Propidium iodide, ECL Kit, DAPI, RNase A, and MTT reagents were purchased from Sigma-Aldrich. DMEM medium and fetal bovine serum (FBS) was purchased from Invitrogen, USA.

### Cell line and culture conditions

MDA-MB-231 and MDA-MB-468 breast cancer cells were purchased from National Centre for Cell Science, Pune, India. Cells were grown in T25 flask using DMEM complete medium. After 70% confluence cells were passaged regularly and kept in humidified 5% CO_2_ incubator. For each experiment cells were taken from T25 flask.

### Generation of resistant cell lines

Docetaxel resistant MDA-MB-231 and MDA-MB-468 cells were generated from sensitive cell lines according to earlier reported method with some modifications^39^. Initially, sensitive MDA-MB-231 and MDA-MB-468 cells were treated with a sub-lethal dose of docetaxel (20 nM) for 48 hr. Then, docetaxel containing medium was discarded to remove dead floating cells and fresh medium was added for 72 hr to recover the live cells. Cells were then passaged. Live cells were again treated with 40 nM docetaxel for 48 hr and after that cells were kept in fresh medium for 72 hr. In this way we increased the dose by 20 nM and ultimately around 1000 nM docetaxel was used to treat the cells. At the end of one year time, cells became resistant (MDA-MB-231R and MDA-MB-468R). In these cells, MTT assay was performed to access drug sensitivity.

### MTT assay

Anti-proliferative effect was determined by colorimetric based MTT assay according to earlier reported method^40^. In brief, MDA-MB-231, MDA-MB-468, MDA-MB-231R and MDA-MB-468R cells were seeded in 96 well plates. After overnight incubation, cells were treated with different concentration of docetaxel for 48 h. After treatment, medium was discarded and MTT solution (1 mg/ml in incomplete DMEM medium) was added and cells were incubated for 5 h. After incubation MTT solution was discarded and DMSO was added in each wells.

### Phase contrast microscopy

MDA-MB-231, MDA-MB-468, MDA-MB-231R and MDA-MB-468R cells were seeded in 30 mm Petri dish. The morphology of sensitive as well as resistant cells was captured by phase contrast microscopy^41^. In another set of experiment, MDA-MB-231R and MDA-MB-468R cells were seeded in 30 mm Petri dish. After 70% confluence, cells were treated with Iturin A and/or docetaxel for 48 h. The dose of Iturin A was 4 µM and 5.6 µM Iturin A for MDA-MB-231R and MDA-MB-468R respectively. The dose of docetaxel was 50 nM for both resistant cell lines. After treatment, phase contrast images of treated resistant cells were captured by microscope. In next experiments, resistant cells were treated with above mentioned Iturin A and docetaxel.

### Actin reorganization

Actin reorganization of resistant cells was visualized by rhodamine phalloidin/DAPI staining according to previously reported method^41^. Briefly, MDA-MB-231R and MDA-MB-468R cells were seeded on cover slips. After 70% confluence, cells were treated with Iturin A and/or docetaxel for 48 h. After treatment, cells were washed, fixed and stained with rhodamine phalloidin. Florescent images of treated resistant cells were captured by Zeiss Observer Z1 microscope (Carl Zeiss, Germany) at 20 × magnifications.

### Cell cycle analysis

Quantitative apoptotic effect was determined by DNA content based flow cytometric analysis according to earlier reported method^42^. MDA-MB-231R and MDA-MB-468R cells were seeded in 60 mm Petri dish. Cells were treated with Iturin A and/or docetaxel for 48 h in incomplete medium. After treatment, cells were harvested by trypsin treatment. Collected cells were fixed in 70% chilled ethanol for overnight. Then, cells were stained with propidium iodide mixed with RNAse. Cells were kept at 37 °C for 30 minutes. Samples were subjected to flow cytometric analysis.

### Annexin/PI staining

Externalization of phosphatidylserin (Marker of early apoptosis) was checked by Annexin-FITC/PI staining in treated cells according to earlier reported method^43^. Briefly, MDA-MB-231R and MDA-MB-468R cells were seeded in 30 mm Petri dish. Cells were then treated with Iturin A and/or docetaxel for 48 h. After treatment, cells were harvested and cell suspension was stained using Annexin V-FITC apoptosis detection kit (sigma) according to manufacturer protocol. Cells were then placed on glass slides and florescent images were captured by Zeiss Observer Z1 microscope (Carl Zeiss, Germany) at 20 × magnifications.

### Colony formation assay

Cellular reproductive growth of resistance cells were determined by colony formation assay as earlier reported method^29^. Resistant cells were initially treated with Iturin A and/or docetaxel for 24 h. Cells were harvested and 1000 cells were seeded in six well plates. After 7 days, number of colony was counted in each group. Each colony contains greater than 50 cells.

### Western blot analysis

Western blot analysis of MDA-MB-231, MDA-MB-468, MDA-MB-231R and MDA-MB-468R cells was performed to check Akt level according to earlier reported method^44^. In brief, MDA-MB-231, MDA-MB-468, MDA-MB-231R and MDA-MB-468R cells were seeded on 60 mm Petri plats. After 70% confluence cells were collected, washed with PBS and lysed with NP-40 lysis buffer. After lysis cell debris was separated by centrifuge and clear supernatants was collected in fresh tube. Concentration of protein was estimated by commercially available protein estimation kit. Samples were prepared by adding 6x dye and boiling. Equal amount of protein was loaded in each wells and subjected to SDS PAGE gel electrophoresis. Protein was transferred to nitrocellulose membrane. Detection of protein expression was evaluated after using primary and secondary antibodies. In another experimental setup, resistant cells were treated with Iturin A and/or docetaxel. Western blot analysis was performed to evaluate a number of protein expressions.

### Statistical analysis

All experiments were performed at least three times. Experimental values were presented as mean ± S.D. Graphpad Prism software was used to draw bar graphs. P value was calculated using student’s t test. P value < 0.05 was considered statistically significant. P value was calculated in various experiments to compare significance among the various groups.
